# Isolation and Characterization of Lytic Bacteriophages Capable of Infecting Diverse Multidrug-Resistant Strains of *Pseudomonas aeruginosa*: PaCCP1 and PaCCP2

**DOI:** 10.3390/ph17121616

**Published:** 2024-11-30

**Authors:** Boris Parra, Maximiliano Sandoval, Vicente Arriagada, Luis Amsteins, Cristobal Aguayo, Andrés Opazo-Capurro, Arnaud Dechesne, Gerardo González-Rocha

**Affiliations:** 1 Laboratorio de Investigación en Agentes Antibacterianos (LIAA), Departamento de Microbiología, Facultad de Ciencias Biológicas, Universidad de Concepción, Concepción 4070409, Chile; 2 Grupo de Estudio en Resistencia Antimicrobiana (GRAM), Universidad de Concepción, Concepción 4070409, Chile; 3 Facultad de Medicina Veterinaria y Agronomía, Instituto de Ciencias Naturales, Universidad de las Américas, Av. Jorge Alessandri 1160, Campus El Boldal, Concepción 4070409, Chile; 4 Department of Biotechnology and Biomedicine, Technical University of Denmark, Søltofs Plads, Building 221, 2800 Kongens Lyngby, Denmark

**Keywords:** bacteria, bacteriophages, virus, *P. aeruginosa*, antimicrobial resistance, wastewater

## Abstract

Background/Objectives: Antimicrobial resistance (AMR) is a major public health threat, which is exacerbated by the lack of new antibiotics and the emergence of multidrug-resistant (MDR) superbugs. Comprehensive efforts and alternative strategies to combat AMR are urgently needed to prevent social, medical, and economic consequences. *Pseudomonas aeruginosa* is a pathogen responsible for a wide range of infections, from soft tissue infections to life-threatening conditions such as bacteremia and pneumonia. Bacteriophages have been considered as a potential therapeutic option to treat bacterial infections. Our aim was to isolate phages able to infect MDR *P. aeruginosa* strains. Methods: We isolated two lytic phages, using the conventional double layer agar technique (DLA), from samples obtained from the influent of a wastewater treatment plant in Concepción, Chile. The phages, designated as PaCCP1 and PaCCP2, were observed by electron microscopy and their host range was determined against multiple *P. aeruginosa* strains using DLA. Moreover, their genomes were sequenced and analyzed. Results: Phage PaCCP1 is a member of the *Septimatrevirus* genus and phage PaCCP2 is a member of the *Pbunavirus* genus. Both phages are tailed and contain dsDNA. The genome of PaCCP1 is 43,176 bp in length with a GC content of 54.4%, encoding 59 ORFs, one of them being a tRNA gene. The genome of PaCCP2 is 66,333 bp in length with a GC content of 55.6%, encoding 102 non-tRNA ORFs. PaCCP1 is capable of infecting five strains of *P. aeruginosa*, whereas phage PaCCP2 is capable of infecting three strains of *P. aeruginosa*. Both phages do not contain bacterial virulence or AMR genes and contain three and six putative Anti-CRISPR proteins. Conclusions: Phages PaCCP1 and PaCCP2 show promise as effective treatments for MDR *P. aeruginosa* strains, offering a potential strategy for controlling this clinically important pathogen through phage therapy.

## 1. Introduction

*P. aeruginosa* is a Gram-negative, non-glucose-fermenting bacteria that frequently causes opportunistic hospital-acquired infections. It is responsible for infections in various organs, including the skin, lungs, urinary tract, kidneys, and the gastrointestinal tract. Moreover, it is recognized as the most important cause of bacterial infection in cystic fibrosis patients, as the inflammation it triggers contributes to the progression of lung disease [1]. *P. aeruginosa* infects patients with burn wounds, immunodeficiency, chronic obstructive pulmonary disease, cancer, and those with severe infection requiring mechanical ventilation [2]. The symptoms of these infections include generalized inflammation and sepsis, which often require prolonged antimicrobial chemotherapy. If such colonization occurs in critical organs, the outcome can be fatal [3]. *P. aeruginosa* infections are particularly difficult to treat because of its intrinsic resistance to antibiotics [4], and it shows high resistance to a series of commonly used antibiotics, including β-lactams, fluoroquinolones, and aminoglycosides [2]. The number of MDR strains is increasing worldwide [5]. In addition, *P. aeruginosa* produces a variety of virulence factors, including rhamnolipids, pyocyanin, and biofilms [6,7]. The list of priority pathogens compiled by the World Health Organization (WHO) [8] was recently updated [9]. *P. aeruginosa* was reclassified from the critical priority group to the high priority group, but it remains very relevant to global public health, which implies the need to develop new antimicrobials against this pathogen.

Bacterial viruses, known as bacteriophages, have been proposed as a novel therapeutic strategy to control bacterial infections, termed “phage therapy”. Phages offer a promising solution due to their unique antibacterial properties. Although phages were first explored for human therapy shortly after their discovery over a century ago, their use was soon eclipsed by the advent of antibiotics, except in some countries in eastern Europe, like Georgia and its Eliava Institute in Tbilisi [10,11]. Unlike antibiotics, phages are highly specific, targeting only harmful bacteria while leaving beneficial bacteria unharmed. This specificity prevents collateral damage to the healthy commensal microbiota of humans and animals, in contrast to the effects of broad-spectrum antibiotics [12]. In recent years, phage therapy has experienced a resurgence, primarily driven by the increasing issue of AMR [13].

Due to the importance of *P. aeruginosa*, many specific bacteriophages against this pathogen have been described. Most of them are virulent (strictly lytic), tailed, have dsDNA, and belong to the *Caudoviricetes* class [14]. Common receptors targeted by *P. aeruginosa* phages include LPS, OMPs, EPS, pili, and flagella [15]. The exact receptors used can vary between phages and strains they infect. These interactions are highly specific, which allows phages to be selective to the bacterial species and strains they infect. *P. aeruginosa* phages have been isolated from different sources all over the world; nevertheless, it seems that sewage, including hospital and wastewater treatment plant sewage, is the best choice for their isolation [16,17]. Many of these phages have been used in cocktail applications in vitro to evaluate their potential against clinical isolates, including MDR strains in planktonic cultures or biofilms [18]. For instance, it was recently demonstrated that the phage Paride can kill dormant cells, and, in combination with carbapenem or meropenem, eradicated deep-dormant cultures in vitro and reduced a resilient bacterial infection of a tissue cage implant in mice [19]. Moreover, several works have been performed in animals [20,21] and human trials [22,23,24,25,26]. Further research on isolating phages is necessary, as using cocktails with varying host ranges in a single suspension is more effective at inhibiting bacterial infections and reduces the likelihood of bacterial resistance [27].

One of the main mechanisms by which bacteria become resistant to phages is the CRISPR-Cas system [28,29,30]. In *P. aeruginosa,* diverse types of CRISPR-Cas systems have been identified in >30% of the strains, with the number likely to increase as more of these gene families are discovered [31,32,33,34]. On the other hand, there is an evolutionary “arms race” between phages and bacteria. To overcome the bacterial protection afforded by CRISPR-Cas systems and promote their own replication, phages have developed Anti-CRISPR systems (Acr proteins) [35,36].

Our aim was to isolate lytic phages able to infect multiple strains of the relevant pathogen *P. aeruginosa* strains. In this work, we describe phages PaCCP1 and PaCCP2, which were isolated from wastewater in Concepción, Chile. These phages belong to distantly related genera in the class *Caudoviricetes*. They can infect and propagate in diverse MDR *P. aeruginosa* strains.

## 2. Results and Discussion

### 2.1. Phage Characterization

**Figure 1:**
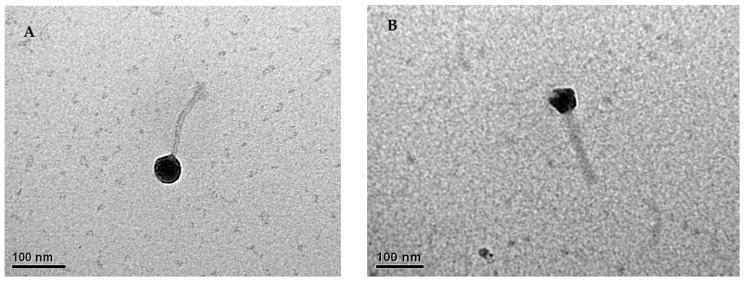
Transmission electron micrographs of phages PaCCP1 (**A**) and PaCCP2 (**B**). Images show the head and tail of both phages.

Two lytic phages able to infect *P. aeruginosa* strains were isolated from wastewater samples. The isolated phages, designated as PaCCP1 and PaCCP2, produced circular plaques with a diameter of 2–3 mm in DLA. Electron microscopy revealed that they are tailed-containing phages (Figure 1). Phage PaCCP1 has a capsid head of 53 nm (±4 nm) and a tail of 157 nm (±8 nm), while phage PaCCP2 has a capsid head of 46 nm (±5 nm) and a tail of 154 nm (±6 nm).

**Figure 2:**
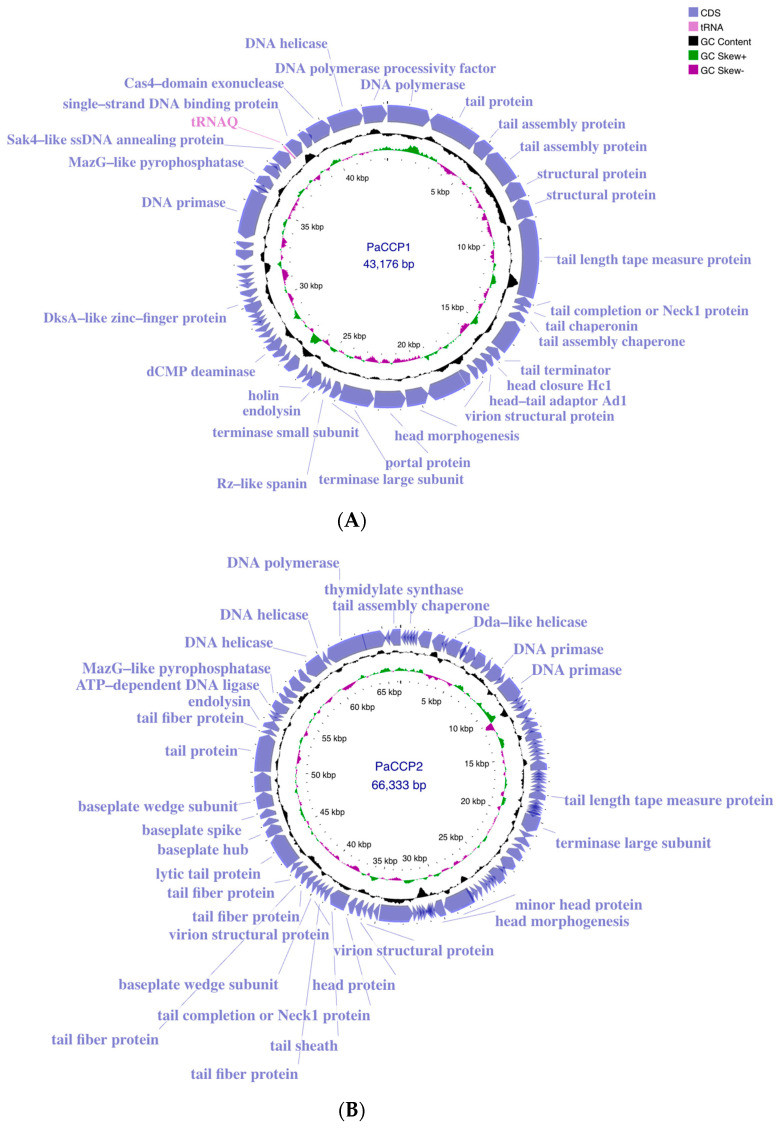
Genome maps of phages PaCCP1 (**A**) and PaCCP2 (**B**) obtained using Proksee. ORFs with identified functions are shown. Moreover, GC content and GC skew are indicated.

**Figure 3:**
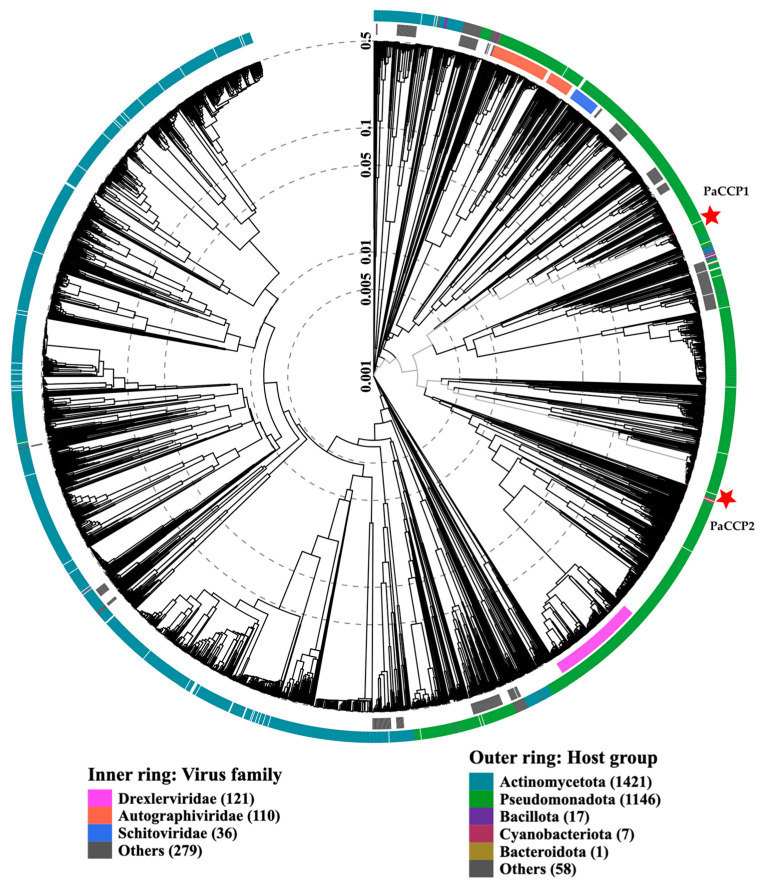
ViPTree analysis of *Pseudomonas* phages PaCCP1 and PaCCP2. Phages are identified according to their official ICTV classification, with the outer and inner rings indicating their host group and virus family, respectively.

The genome of the phage PaCCP1 consists of a dsDNA molecule, 43,176 bp in length, with a GC content of 54.4% (accession number PQ492277) (Figure 2). Its relative phages belong to the *Septimatrevirus* genus, formerly known as *Septima3virus*, which was created in 2015 by ICTV in proposal 2015.054a-dB. Originally, the genus included 5 species, but currently includes 21 species (ICTV proposal 2023.039B). According to the latter proposal, this genus was incorporated in the new subfamily *Jondennisvirinae*, which contains three genera (*Septimatrevirus, Kipunavirus, Kilunavirus*) and belongs to the class *Caudoviricetes* without belonging to a specific family (Figure 3), after the abolishment of morphology-based taxa in 2022 by ICTV [37]. To explore the taxonomy of phage PaCCP1, we downloaded all the *Septimatrevirus* genomes according to ICTV from GenBank in July 2024.

It is relevant to notice that some *Septimatrevirus* phages can infect bacteria that belong to genera other than *Pseudomonas* (Table 1). For instance, phage Samson and DoCa1 can infect plant-associated *Xanthomonas* strains. Phages DLP1 and DLP2 can infect *Stenotrophomonas maltophilia* strains. Interestingly, phages DLP1 and DLP2 also can infect *Pseudomonas* strains showing a cross-taxonomic order infectivity [38].

The genome of the phage PaCCP2 consists of a dsDNA molecule, 66,333 bp in length and with a GC content of 55.6% (accession number PQ492278) (Figure 2). Their relatives belong to genus *Pbunavirus,* formerly known as *Pb1likevirus* and subsequently renamed *Pbunalikevirus*. The genus, created in 2009 by ICTV (proposal 2009.001a-gB), belongs to the class *Caudoviricetes* without belonging to a specific family or subfamily (Figure 3). Originally, the genus comprised seven phages, but it currently includes 32 species (ICTV proposal 2021.061B). Originally, the genus included six *Pseudomonas* phages and one *Burkholderia* phage (BcepF1); however, in 2020, the genus *Bcepfunavirus* was created with phage BcepF1 (ICTV proposal 2020.116B). *Pbunavirus* is one of the most rapidly growing *Pseudomonas* myovirus genera (ICTV proposal 2021.061B). Phage PB1 is the type species (the phages within the genus are also known as PB1-like) and it was first isolated and described almost half a century ago by Holloway et al. [47] from sewage samples. *Pbunavirus* is ubiquitous on Earth, and is found in the US, Europe (France, Portugal, Poland, Spain, Russia, Germany, the Netherlands, and Scotland), and Brazil in soil, freshwater, wastewater, and activated sludge samples [48,49]. *Pbunavirus* includes phages that can infect only *P. aeruginosa* strains [49]. To explore the taxonomy of phage PaCCP2, we downloaded the genomes of all the phages described as *Pbunavirus* according to ICTV in July 2024 (Table 2).

According to ICTV’s Bacterial and Archaeal Viruses Subcommittee, two phages are assigned to the same species if their genomes are more than 95% identical at the nucleotide level over their full genome length, tested reciprocally [62]. The Subcommittee also established a 70% nucleotide identity of the full genome length as the cutoff for genera, calculated in the same way as the species cutoff. These values can be calculated by several tools, such as BLASTn (% identity multiplied by % coverage) or VIRIDIC [63]. According to Simmonds et al. [64], these values only serve as an approximation of evolutionary relatedness and the relationship among viruses should be explored by phylogenetic methods that are also capable of calculating clade support, such as VICTOR [65]. We used VICTOR because it was specifically designed for prokaryotic viruses and can output classification at the species, genus, and family ranks. Moreover, we compared the VICTOR and VIRIDIC outputs.

**Figure 4:**
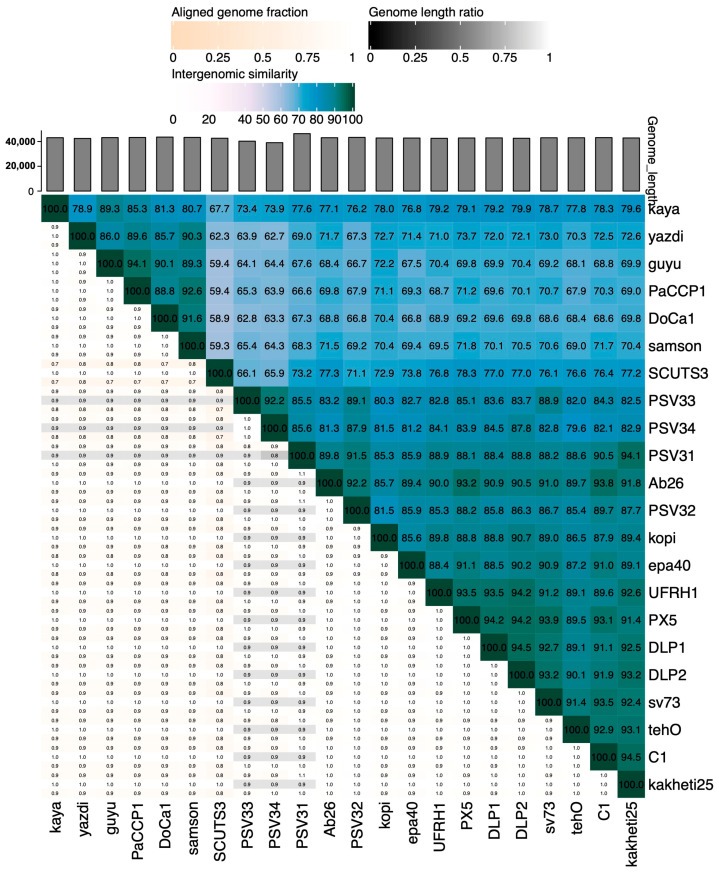
Heatmap showing the intergenomic similarities of phage PaCCP1 and *Septimatrevirus* phages.

**Figure 5:**
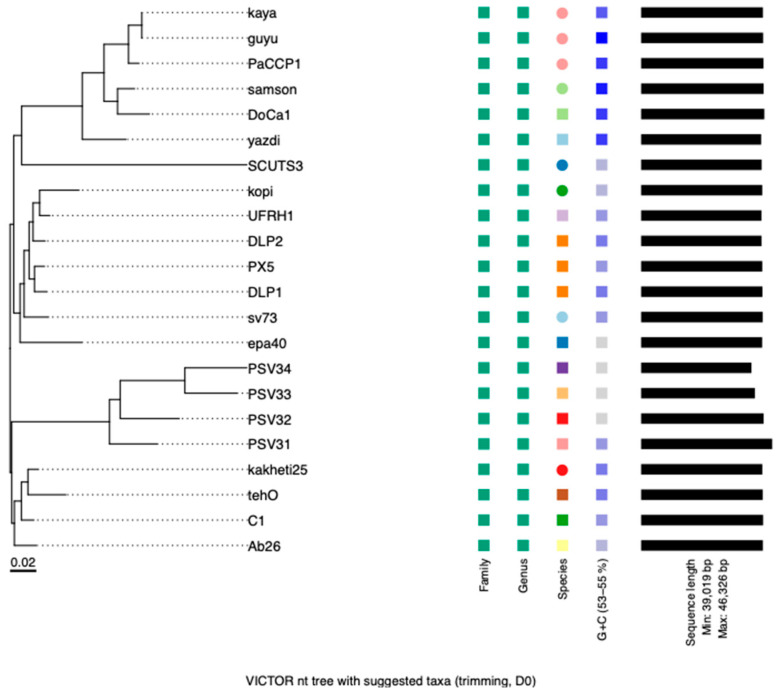
Phylogenetic analysis of phage PaCCP1 and *Septimatrevirus* phages. The scale bar indicates the number of substitutions per site. Each species is indicated by a unique color.

According to VIRIDIC, PaCCP1 belongs to a unique species and, together with Guyu, Samson, DoCa1, yazdi, and kaya, forms another genus different from *Septimatrevirus* (Figure 4). However, according to VICTOR, phages PaCCP1, Guyu, and Kaya belong to the same species and there is not a new genus, because all the *Septimatrevirus* phages and PaCCP1 belong to the same genus (Figure 5). Therefore, we assume the latter classification. Interestingly, our VICTOR analysis indicated that phages DLP1, DLP2, and PX5 belong to the same species.

**Figure 6:**
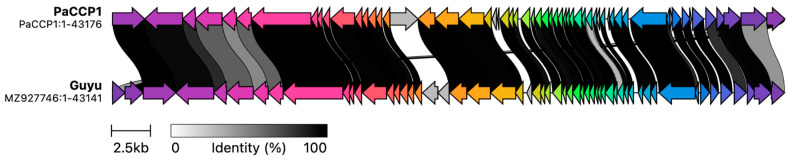
Comparison of phage PaCCP1 and its closest relative, phage Guyu, using alignment of all annotated proteins. The arrow’s colors represent the gene clusters encoding similar proteins. The lines linking the arrows show gene-encoding proteins that share more than 80% sequence identity.

The genome annotation of phage PaCCP1 demonstrated that it encodes 59 ORFs, 46 (78%) of which are encoded on the positive strand and 13 (22%) on the negative strand. One ORF encodes a tRNA (trnQ). Twenty-eight ORFs (46.7%) were identified as hypothetical proteins with unknown functions. The functional ORFs include a minor and a major capsid protein, two terminase proteins, a scaffolding protein, three tail proteins, and one exonuclease. No genes associated with bacterial virulence or AMR were identified, and the lifestyle of PaCCP1 was classified as lytic. The proteome structure is very similar to their closest relative, phage Guyu (Figure 6).

No further characterization of Guyu and Kaya was performed, such as host range or morphology observed by electronic microscopy. Both phages were isolated from a river in Haining, China, in 2020 using the strain *P. aeruginosa* PAO1 PA2072 [39]. The Guyu genome is 43,141 bp long, with a CG content of 56%, and it encodes 56 proteins, while the Kaya genome is 43,067 bp in length with a CG content of 54% and encodes 60 proteins. Phage samson, another phage related to PaCCP1, is a *Xanthomonas* phage that lacks phenotypic characterization. It was isolated from sewage samples from Texas, and contains a 43,314 bp genome with 58 predicted genes [40].

A phage named TR (accession number OL802211) described by Xuan et al. [66] was isolated from sewage samples in Qingdao, China, using *P. aeruginosa* PAO1. It is not classified as *Septimatrevirus* yet, but it probably will be. According to VIRIDIC, it showed an intergenomic similarity of 91,8 with PaCCP1. It was observed by electron microscopy, showing that it contains a head of approximately 50 nm with a long noncontractile tail of approximately 170 nm. Its genome is 43,354 bp long, with a CG content of 55%, and it encodes 56 proteins. It was demonstrated that the type IV pilus (TP4) acts as an adsorption receptor for phage TR. In this regard, it has been demonstrated that T4P is not only the virulence factor for some pathogens but is also the receptor for many *P. aeruginosa* phages [66]. Recently, Su et al. [67] described the host range of phage TR against thirteen *P. aeruginosa* strains using the visual assessment of plaques on the spot test. They indicated that six (46.2%) of the *P. aeruginosa* strains tested were lysed by phage TR.

**Figure 7:**
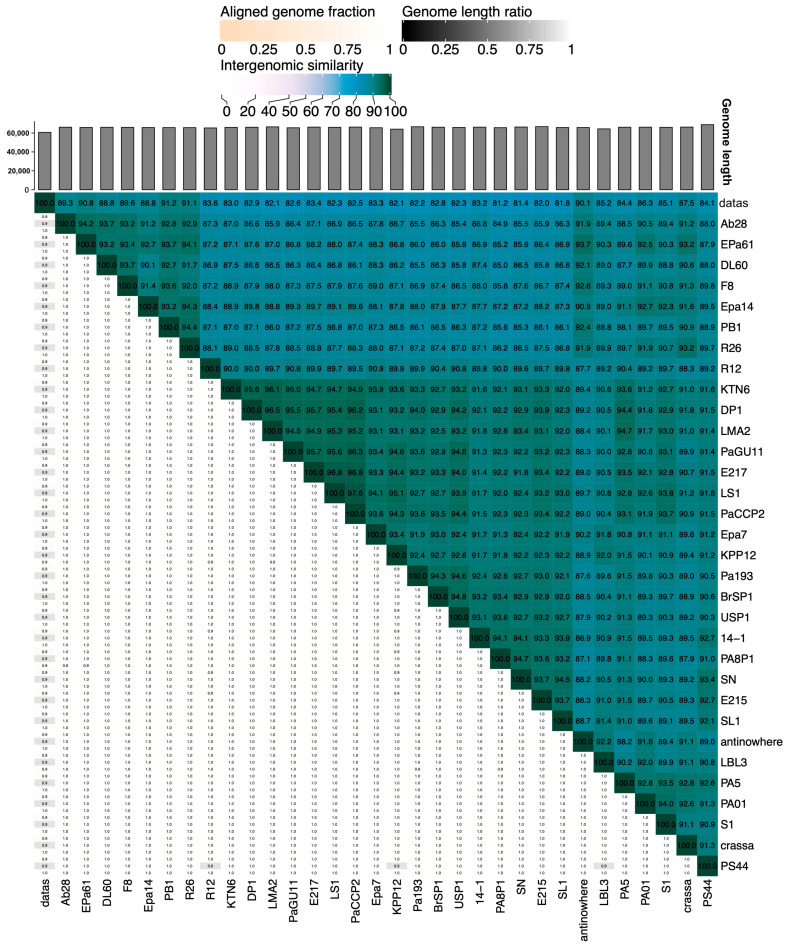
Heatmap of the intergenomic similarities of phage PaCCP2 with *Pbunavirus* phages.

**Figure 8:**
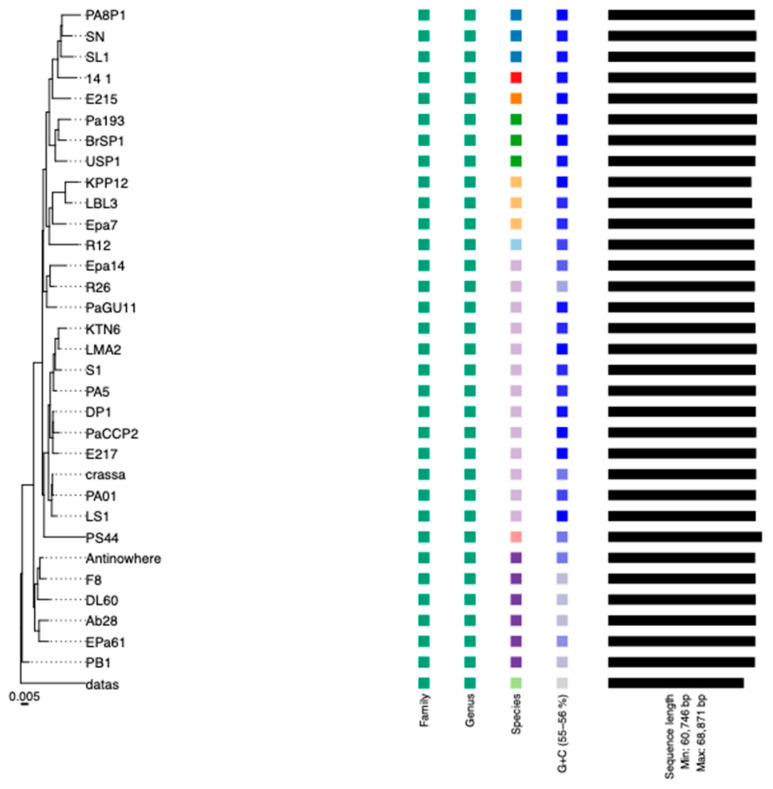
Phylogenetic analysis of phage PaCCP2 and *Pbunavirus* phages. The scale bar indicates the number of substitutions per site. Each species is indicated by a unique color.

According to VIRIDIC, phage PaCCP2 belongs to the same species as phages LS1, E217, and PaGU11 in the *Pbunavirus* genus (Figure 7). However, according to VICTOR, it belongs to the same species as epa14, R16, PaGU11, KTN6, LMA2, S1, PA5, DP1, E217, crassa, Pa01, and LS (Figure 8). Some of these phages have been characterized. For instance, it has been demonstrated that phage LS1 (*Pbunavirus*) has high inhibition potential on the growth of *P. aeruginosa* biofilm formation [50].

**Figure 9:**
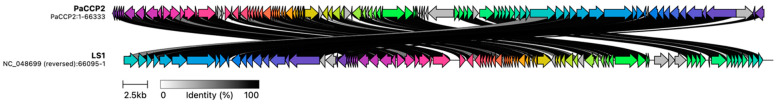
Comparison of phage PaCCP2 and its closest relative, phage LS1, using alignment of all annotated proteins. The arrow’s colors represent the gene clusters encoding similar proteins. The lines linking the arrows show gene-encoding proteins that share more than 80% sequence identity.

The genome annotation of phage PaCCP2 demonstrated that it encodes 106 ORFs, 43 (40.6%) of which were detected on the positive strand and 63 (59.4%) on the negative strand. Only 31 ORFs (29.2%) were annotated as functional proteins. The functional ORFs include, among others, two DNA helicases, a DNA polymerase, a large terminase subunit, eleven tail-related proteins, and an endolysin protein. In this regard, it has been described that virion particles of *Pbunavirus* are composed of at least 22 different proteins [52,68]. No genes associated with bacterial virulence or AMR were identified, and the lifestyle of PaCCP2 was categorized as lytic. The proteome structure is very similar to their closest relative, phage LS1 (Figure 9).

Several *Pbunavirus* phages are part of a phage cocktail developed to eradicate *P. aeruginosa* infections. For instance, Pa193 was part of a phage cocktail candidate developed by Armata Pharmaceuticals [56,69]. For instance, Forti et al. [51] demonstrated that a phage cocktail composed of six phages (PYO2, DEV, E215 and E217, PAK_P1 and PAK_P4) was able to lyse *P. aeruginosa* both in planktonic liquid cultures and in biofilms. From these phages, E217 is a *Pbunavirus*.

Wannasrichan et al. [70] demonstrated that *P. aeruginosa* strains resistant to phage JJ01 (*Pbunavirus*) exhibit hypersensitivity to colistin and reduce biofilm production. This trade-off has been broadly demonstrated [71]. This is helpful for phage therapy, because if bacteria become phage-resistant, they become less virulent at the same time.

### 2.2. Inhibition Assay and Host Range

We demonstrated that the addition of phages PaCCP1 or PaCCP2 at MOI 10 after 6 h of incubation effectively prevented the growth of *P. aeruginosa* UC535 or UC550, respectively.

Our experiments, performed to evaluate the host range of phages PaCCP1 and PaCCP2, indicate that even though phage PaCCP1 was isolated with strain UC528, it can successfully infect and propagate in strains UC522, UC532, UC533, and UC536 in DLA. On the other hand, phage PaCCP2, isolated with strain UC335, successfully infects and propagates in strains UC522, UC529, and UC532 in DLA.

Even though all the strains were negative for carbapenemase production using Blue Carba, it is relevant to notice that both phages PaCC1 and PaCCP2 can infect and propagate in strain UC532 (Table 3), which is an MDR strain resistant to amikacin, ciprofloxacin, meropenem, and imipenem. Strains UC529 and UC532 can also be categorized as MDR because they showed non-susceptibility to at least one agent in three antimicrobial categories [72]. These results demonstrated the effectiveness of our isolated phages, PaCCP1 and PaCCP2, against *P. aeruginosa* and therefore make them a very attractive alternative option to antibiotics or for use in combination with antibiotics or other antimicrobials for improved performance.

### 2.3. Anti-CRISPR Proteins in Phages PaCCP1 and PaCCP2

As far as we know, Acrs have not been searched for in any previous work on *Septimatrevirus* and *Pbunavirus*. Using two tools for cross validation, PaCRISP [73] and AcRanker [74], we identified several putative Acr-encoding genes in both phages PaCCP1 and PaCCP2 (Table 4). Both tools are machine learning-based programs.

In phage PaCCP1, seven candidate genes were identified using PaCRISP and five using AcRanker. Merging the results of both predictors, we suggested that CDS 41, 33, and 28 could be Acr-encoding genes because these genes were identified using both tools, were ranked in the first three places in the results obtained with AcRAnker (Table 4), and encode for small proteins (69, 101, and 69, respectively), which is a typical size for Acrs, usually between 50 and 150 amino acids [35]. In addition, all of these genes encode hypothetical proteins without predicted function after functional annotation performed using PHANOTATE [75], a CDS-prediction tool specifically designed for phages, in the Pharokka [76] and PHROGs database [77]. In phage PaCCP2, seventeen candidate genes were identified using AcRanker and nineteen using PaCRISP. CDS 39, 43 73, 35, 36, and 1 were identified in both tools. From them, only CDS 73 has a putative function assigned (head and packaging), and it is the largest one.

It has been described that Acrs proteins do not usually share conserved sequences; therefore, their discovery is sometimes difficult [78]. Anti-CRISPR systems have been described in *P. aeruginosa* phages [35,79,80], but they have not been studied in detail and have only been investigated in a few of the newly described phages [78]. The validation of the candidate Acr-encoding genes detected in phages PaCCP1 and PaCCP2 needs protein expression, purification, and biochemical characterization, which we are considering for future experiments in new projects.

## 3. Materials and Methods

### 3.1. Bacterial Strains

We used *P. aeruginosa* strains provided by the clinical laboratories of tertiary hospitals in 2020, with no direct involvement of patients in the study. The isolates were preliminarily identified as *Pseudomonas* spp. using biochemical and physiological tests, colony morphology, and pigment production. Gram-negative bacilli with the above-mentioned characteristics were considered as *P aeruginosa* based on a positive oxidase test, a triple sugar iron agar reaction of alkaline over no change, growth at 42 °C, and the production of bright blue to blue green diffusible pigment on Mueller–Hinton agar (Thermo Fisher Scientific, Waltham, MA, USA).

Strain UC528 was used to isolate the phage PaCCP1. This bacterial strain was obtained from a skin wound of a patient at a hospital in the city of Concepcion (Chile). Strain UC536 was used to isolate the phage PaCCP2. This bacterial strain was obtained from a urine sample of a patient hospitalized in a hospital located in the city of Talca (Chile). Moreover, 20 clinical strains of *P. aeruginosa* obtained from hospitalized patients from the same hospitals described above were used to determine the host range. We included *P. aeruginosa* reference strains obtained from the American Type Culture Collection (ATCC) (Manassas, VA, USA): ATCC 27853, ATCC 9027, and PAO1. All bacterial strains were stored in Luria–Bertani (LB) broth (Thermo Fisher Scientific, Waltham, MA, USA) containing 20% glycerol (Thermo Fisher Scientific, Waltham, MA, USA), kept at −80 °C, and were routinely grown in LB media at 37 °C.

### 3.2. Sample Collection and Processing

Samples from a municipal wastewater treatment plant (WWTP) in Concepción, Chile, were collected in January 2024. They consisted of 1 L of 24 h composite water from the influent of the WWTP, prior to any treatment. The samples were kindly provided by the Centinela Biobío-UCSC Center, a wastewater monitoring center of the Universidad Católica de la Santísima Concepción, Chile. Samples were collected in sterile 50 mL tubes (Corning, Glendale, AZ, USA) and stored at 4 °C until use within 2 days. The samples were centrifuged at 8000× *g* for 45 min at 4 °C, and the supernatant was collected to remove large particles and most bacterial cells. To remove the remaining bacterial debris while retaining the viral fraction, the supernatant was passed through a sterile 25 mm Whatman glass fiber membrane (MilliporeSigma, Burlington, MA, USA) with a pore size of 0.22 µm.

### 3.3. Disk Diffusion Method for the Determination of Antimicrobial Resistance

All strains were tested for carbapenemase production using Blue Carba [81], and antibiotic susceptibility was determined using the disk diffusion method in Mueller–Hinton agar according to the Clinical and Laboratory Standards Institute (CLSI) [82]. The zones of growth inhibition around each of the antibiotic disks were measured and related to the susceptibility of the isolate and to the diffusion rate of the drug through the agar medium (Thermo Fisher Scientific, Waltham, MA, USA). The zone diameters of each drug were interpreted using the criteria published by the CLSI. The test was performed using the following antibiotics: amikacin (AMK), ciprofloxacin (CIP), meropenem (MEM), imipenem (IPM), ceftazidime/avibactam (CZA), ceftazidime (CAZ), cefepime (FEP), and piperacillin/tazobactam (TZP) (Thermo Fisher Scientific, Waltham, MA, USA). 

### 3.4. Phage Isolation

The phages were isolated using the classical DLA method [83]. Overnight cultures of 20 *P. aeruginosa* strains were prepared in LB broth and incubated at 37 °C. An aliquot of 100 µL from the overnight culture was inoculated into fresh LB broth and incubated at 37 °C until the mid-exponential phase was reached. Aliquots (100 µL) of each bacterial suspension were mixed with 100 μL of filtered environmental samples and 3 mL of melted (50 °C) soft LB agar (0.5%) supplemented with CaCl_2_ (final concentration 5 mM) (Thermo Fisher Scientific, Waltham, MA, USA). After overnight incubation of the plates at 37 °C, single plaques were picked and harvested in 500 µL of SM buffer (100 mM NaCl, 8 mM MgSO_4_, and 50 mM Tris-HCl, pH 7.5) (Thermo Fisher Scientific, Waltham, MA, USA). The isolates were then purified three times by DLA and sequential isolation.

### 3.5. Susceptibility to RNAse and Chloroform

The susceptibility of the isolated phages to RNAse was determined by DLA after the addition of RNAse (final concentration10 µg mL^−1^) to the bottom agar. The resistance of the isolates to chloroform (Thermo Fisher Scientific, Waltham, MA, USA) was determined by adding it to 200 µL of phage suspensions at a final concentration of 10 or 100 µL mL^−1^. The mixtures were then incubated for 60 min at room temperature and phage viability was assessed using DLA. All assays were performed in triplicate.

### 3.6. Propagation and Concentration of Phages

Concentrated stocks of phages were obtained using Amicon ultrafiltration membranes (100 kDa) (MilliporeSigma, Burlington, MA, USA). Phage suspensions were mixed with logarithmic growing cultures of each host strain, as described previously. Phages were then added at a multiplicity of infection (MOI) of 0.01 in 15 mL bacterial cultures. Samples were incubated overnight at 37 °C with shaking at 120 rpm. After incubation, the suspensions were centrifuged and filtered to remove bacterial debris and then transferred to Amicon and centrifuged at 3000× *g* for 20 min at 4 °C or until a remaining volume of less than 1 mL was achieved. The titer of the phage suspensions was determined by DLA, as described previously, and expressed as plaque-forming units per milliliter (PFU mL^−1^).

To obtain more concentrated stocks, precipitation with polyethylene glycol 8000 (PEG) (Merck, Rahway, NJ, USA) was performed. Briefly, phage suspensions and bacterial cultures were mixed, incubated overnight at 37 °C, filtered, and precipitated with 10% PEG and 1 M NaCl (Merck, Rahway, NJ, USA). The mixtures were then incubated at 4 °C overnight and centrifuged for 1 h at 16,000× *g* to obtain a phage pellet, which was resuspended in 1 mL of SM buffer. The titer of the phage suspensions was determined using DLA, as described previously.

### 3.7. Electron Microscopy

We visualized the morphology of the isolated phages using an aliquot of 15 µL from pure suspensions with glow-discharged 200 mesh copper-coated grids. Phages on the grids were incubated for 30 s before blotting off the liquid using a Whatman filter pmembrane (MilliporeSigma, Burlington, MA, USA). The suspensions were then fixed with 5 µL of glutaraldehyde (Merck, Rahway, NJ, USA), incubated for 10 s, and excess liquid was blotted off. The samples were stained with 3 µL of 2% uranyl acetate (Merck, Rahway, NJ, USA) and incubated for 30 s. The microscope used was a JEM 2011 (Jeol, Tokyo, Japan) at Centro de Espectroscopía y Microscopía (CESMI) at Universidad de Concepción. Images were analyzed using ImageJ software v1.54i to calculate the length of their tail and capsid using three particles for each phage.

### 3.8. DNase and RNase Treatment of Phage Stocks

For the effective purification of phage DNA, it was necessary to first remove bacterial DNA and RNA debris. For this, 447 µL of each purified phage suspension was mixed with 50 μL DNase buffer, 3 μL DNase (AMPD1–1KT) (Sigma-Aldrich, Burlington, MA, USA), and 3 μL RNase suspension at 5 mg/mL. The samples were then incubated for 1 h at 37 °C. DNase was inactivated by adding 10 μL DNase stop solution and 40 μL 50 mM EDTA (Thermo Fisher Scientific, Waltham, MA, USA). The titer of phage stocks was determined using DLA after appropriate dilutions.

### 3.9. Nucleic Acid Extraction

DNA extraction was performed from highly concentrated phage stocks (10^11^ PFU mL^−1^) using a Purelink Viral DNA/RNA mini kit (Invitrogen, Thermo Fisher Scientific, Waltham, MA, USA), according to the manufacturer’s instructions. Briefly, samples were treated with Proteinase K (50 μL at 20 mg/mL) for 1 h at 56 °C to break down viral capsid and release phage DNA. The DNA was purified using a column and washed twice with ethanol. The starting material was 0.5 mL, and the elution volume was 50 µL.

Purified DNA was visualized using 0.8% agarose gel electrophoresis. The concentration was quantified using a Qubit 1× dsDNA High Sensitivity Assay Kit (Thermo Fisher Scientific) and a Nanodrop spectrophotometer (Thermo Fisher Scientific) and was stored at −20 °C.

### 3.10. Sequencing

Sequencing was performed at SeqCenter Inc. (Pittsburgh, PA, USA). Illumina sequencing libraries were prepared using a tagmentation-based and PCR-based Illumina DNA Prep kit (Illumina, San Diego, CA, USA) with custom IDT 10 bp unique dual indices (UDIs) with a target insert size of 280 bp. No additional DNA fragmentation or size-selection steps were performed. Illumina sequencing was performed on an Illumina NovaSeq X Plus sequencer in one or more multiplexed shared-flow-cell runs, producing 2 × 151 bp paired-end reads.

### 3.11. Assembly of Genomes

Demultiplexing and adapter trimming were performed using BCL-convert v4.2.4 (Illumina). The reads were quality checked using FastQC v0.12.1 [84]. Assemblies were performed *de novo* using SPAdes v3.14 [85] as recommended by [86] at Phage Galaxy (Center for Phage Technology at Texas A&M University, TX, USA) [87,88]. Assemblies were evaluated using Quast v5.2.0 [89]. The first assemblies generated multiple contigs of diverse sizes with low coverage, indicating bacterial DNA contamination and the need for subsampling. Therefore, reads were randomly subsampled as recommended by Shen et al. [90] using seqtk v1.4 [91] to obtain 2% of the total reads (Table 5).

Assemblies were repeated at the Bacterial and Viral Bioinformatics Resource Center (BV-BRC) [92] using SPAdes. The outputs were visually checked using Bandage v0.8.1 [93], error correction was performed using Pilon v1.24 [94], and Quast was used to check the assemblies. The outputs were a large contig with high coverage (>90) and several short contigs with low coverage (<2) per phage, indicating the assembly of a complete phage genome [90]. Contigs with low coverage corresponding to bacterial DNA contamination were removed manually. High-quality assemblies were further used for annotation and analysis.

### 3.12. Comparative Genome Analysis

The similarity of phages PaCCP1 and PaCCP2 to other described viral genomes was determined using Blastn, considering only complete viral genomes and using default settings. Similar phage sequences were downloaded in July 2024 from GenBank and used as references for further analysis. Pairwise comparisons of the nucleotide sequences were conducted using VIRIDIC [63] to determine the intergenomic similarity using the average nucleotide identity (ANI) obtained by Blastn, based in the classification guidelines described by the International Committee on Taxonomy of Viruses (ICTV) [37,95].

To classify phages and construct phylogenetic trees, Virus Classification and the Tree Building Online Resource (VICTOR) web service was used (https://ggdc.dsmz.de/victor.php, accessed on 20 October 2024) [65]. The similarities and relationships between PaCCP1, PaCCP2, and other reported prokaryotic double-stranded DNA viruses, were analyzed using VIPTree v4.0 [96].

### 3.13. Genome Annotation 

PhaTYP 1.0 [97] and PhageAI 1.0 [98] were used for bacteriophages’ lifestyle prediction. Open reading frames (ORFs) were predicted with PHANOTATE [75] in Pharokka v1.3.2 [76] using the PHROG database (https://phrogs.lmge.uca.fr/, accessed on 20 October 2024) [77] with MMseqs2 v 15-6f452 [99]. Moreover, the annotation of the genomes of phages PaCCP1 and PaCCP2 was performed using the Phage Commander application 1.0 [100]. This tool runs nine gene identification programs such as RAST v.1.9.5 [101], GeneMarks v.4.30 [102], Prodigal v 2.6.3 [103], and Glimmer v3.02 [104]. tRNAs were predicted using ARAGORN v2.36 [105].

Genome maps of both phages PaCCP1 and PaCCP2 were obtained using Proksee v1.0 [106]. Clinker v0.0.26 [107] was used for the visual comparison between PaCCP1 or PaCCP2 with their closest relatives.

### 3.14. Detection of AMR and Virulence Genes

The search of genes encoding antibiotic resistance factors was performed using Resfinder 4.0 [108] and the Resistance Gene Identifier on the Comprehensive Antibiotic Resistance Database (CARD) v1.0, which is a recent tool curated using machine learning [109]. Moreover, the web tool VirulenceFinder 2.0 [110] was used for the search of genes potentially coding for virulence factors (98% ID threshold).

### 3.15. Detection of Anti-CRISPR-Cas Systems

To identify putative Acrs, we used PaCRISPR v1.0 [73] and AcRanker v1.0 [74], which are both tools with a cutoff by default. These programs allow for the direct prediction of Acr-encoding genes de novo with minimal knowledge a priori.

### 3.16. Host Range

The host range was determined using twenty clinical strains of *P. aeruginosa* and the three reference strains ATCC 27853, ATCC 9027, and PAO1. For this, mixtures of overnight bacterial cultures and phage suspensions (from a series of four decimal dilutions) were incubated for 20 min and then spotted (20 μL each) onto LB agar in triplicate. The appearance of plaques in the lawn after overnight incubation at 37 °C indicated the ability of the phages to multiply in the bacterial host. When too many plaques to count appeared for the fourth decimal dilution, experiments were performed repeatedly using the next two dilutions.

### 3.17. Growth Inhibition Assay

Mid-exponential-phase cultures of strains UC535 or UC550 were inoculated with the phages PaCCP1 or PaCCP1, respectively, at an MOI 10 in duplicate in 250 mL flasks containing 100 mL of LB broth and incubated at 37 °C without shaking. Samples of 1 mL were taken every 30 min up to 360 min. The absorbance was measured by spectrophotometry at 600 nm using an Spectrophotometer UVISCO V-1200 (Avantor, Radnor Township, PA, USA). Controls without phages were included.

## 4. Conclusions

We report the characterization and the complete genome analysis of two dsDNA lytic *P. aeruginosa* phages. PaCCP1 is a *Septimatrevirus* and PaCCP2 is a *Pbunavirus* belonging to the class *Caudoviricetes*. Both phages were isolated from a wastewater sample obtained from the influent of a wastewater treatment plant in Concepción, Chile.

They have potential as biocontrol agents even against MDR strains of this major human and animal opportunistic pathogen and are responsible for a wide range of diseases, from soft tissue to life-threatening infections. More studies are needed to evaluate, for instance, the use of phage cocktails in combination with antibiotics to control bacteria, the capacity of phages to eradicate biofilms, the development of phage-resistance, and the use of Anti-CRISPR proteins.

## Figures and Tables

**Table 1:** *Septimatrevirus* phages and their intergenomic similarity with PaCCP1. Species and genus clusters are shown.

Phage	IS with PaCCP1	Species Cluster	Genus Cluster	Genome Size	GC Content	No of CDSs	Host	Reference	Accession
PaCCP1	100.0	11	2	43,176	54.4	60	*P. aeruginosa*	This work	PQ492277
guyu	94.1	7	2	43,141	55	54	*P. aeruginosa*	[39]	NC_069743, MZ927746
samson	92.6	17	2	43,314	54.5	57	*Xanthomonas* sp.	[40]	NC_069744, MN062187
yazdi	89.6	22	2	42,439	54.5	55	*P. aeruginosa*	---	NC_069742, LC552830
DoCa1	88.8	5	2	43,553	54.5	54	*Xanthomonas* sp.	[41]	NC_069745, ON911538
kaya	85.3	9	2	43,067	54.5	58	*P. aeruginosa*	[39]	NC_069741, MZ927745
PX5	71.2	16	1	42,828	53.5	60	*P. aeruginosa*	---	NC_069750, OP422637
kopi	71.1	10	1	42,82	53.5	55	*P. aeruginosa*	[39]	NC_069746, OK330455
sv73	70.7	19	1	42,999	53.5	75	*P. aeruginosa*	[42]	NC_007806, DQ163913
C1	70.3	2	1	43,133	53.5	59	*P. aeruginosa*	---	NC_069749, MG897800
DLP2	70.1	4	1	42,593	53.5	58	*Stenotrophomonas maltophilia*	[38]	NC_029019, KR537871
Ab26	69.8	1	1	43,056	53.5	52	*P. aeruginosa*	[43]	NC_024381, HG962376
DLP1	69.6	3	1	42,887	53.5	57	*Stenotrophomonas maltophilia*	[38]	NC_069751, KR537872
epa40	69.3	6	1	42,788	53	58	*P. aeruginosa*	[44]	NC_069747, MT118304
kakheti25	69.0	8	1	42,844	54	58	*P. aeruginosa*	[45]	NC_017864, JQ307387
UFRH1	68.7	21	1	42,567	53.5	57	*P. aeruginosa*	[46]	NC_072810, OQ259603
tehO	67.9	20	1	43,015	53.5	56	*P. aeruginosa*	[39]	NC_069748, OK330456
PSV32	67.9	13	1	43315	53.3	73	*P. aeruginosa*	---	NC_069755, OP712466
PSV31	66.6	12	1	46,326	53.5	86	*P. aeruginosa*	---	NC_069754, OP712460
PSV33	65.3	14	1	40,244	53.2	71	*P. aeruginosa*	---	NC_069752, OP712474
PSV34	63.9	15	1	39,019	53.3	63	*P. aeruginosa*	---	NC_069753, OP712479
SCUTS3	59.4	18	3	42,622	53.5	62	*P. aeruginosa*	---	NC_072809, MK165657

**Table 2:** *Pbunavirus* phages and their intergenomic similarity with PaCCP2. Species and genus clusters are shown.

Phage	IS with PaCCP2	Species Cluster	Genus Cluster	Genome Size	GC Content	No of CDSs	Host	Reference	Accession
PaCCP2	100.0	10	1	66,333	55.6	102	*P. aeruginosa*	This work	PQ492278
LS1	97.6	10	1	66,095	55.5	93	*P. aeruginosa*	[50]	NC_048699, MG897799
E217	96.8	10	1	66,291	55.5	94	*P. aeruginosa*	[51]	NC_042079, MF490240
PaGU11	96.3	10	1	65,554	55.5	90	*P. aeruginosa*	--	NC_050145, AP018815
DP1	96.3	8	1	66,158	55.5	92	*P. aeruginosa*	[14]	NC_041870, KR869157
LMA2	95.2	8	1	66,530	55.5	94	*P. aeruginosa*	[52]	NC_011166, FM201282
KTN6	94.9	8	1	65,994	55.5	91	*P. aeruginosa*	[53]	NC_041865, KP340288
USP1	94.4	28	1	65,918	55.5	87	*P. aeruginosa*	[54]	NC_050149, MT491204
KPP12	94.3	15	1	64,144	55.5	88	*P. aeruginosa*	[21]	NC_019935, AB560486
S1	93.8	25	1	66,086	55.5	94	*P. aeruginosa*	[55]	NC_048745, MK340760
Pa193	93.6	18	1	66,657	55.5	92	*P. aeruginosa*	[56]	NC_050148, MK837009
Epa7	93.6	13	1	65,629	55.5	94	*P. aeruginosa*	[44]	NC_050146, MT118289
BrSP1	93.5	4	1	66,189	55.5	94	*P. aeruginosa*	---	NC_048675, MF623055
E215	93.4	9	1	66,789	55.5	95	*P. aeruginosa*	[51]	NC_042080, MF490241
PA5	93.1	19	1	66,182	55.5	101	*P. aeruginosa*	[57]	NC_041902, KY000082
SN	92.3	27	1	66,390	55.5	92	*P. aeruginosa*	[52]	NC_011756, FM887021
PA8P1	92.3	20	1	65,690	55.5	93	*P. aeruginosa*	---	NC_048806, MN131142
SL1	92.2	26	1	65,847	55.5	91	*P. aeruginosa*	[58]	NC_048676, MF768470
PA01	91.9	17	1	66,220	55.5	92	*P. aeruginosa*	---	NC_048626, AP019535
PS44	91.5	22	1	68,871	55.5	97	*P. aeruginosa*	---	NC_028939, KM434184
141	91.4	1	1	66,235	55.5	90	*P. aeruginosa*	[52]	NC_011703, FM897211
crassa	90.9	5	1	66,295	55	92	*P. aeruginosa*	---	NC_050151, MT119377
LBL3	90.4	16	1	64,427	55.5	88	*P. aeruginosa*	[52]	NC_011165, FM201281
Epa14	89.6	11	1	65,797	55.5	93	*P. aeruginosa*	[44]	NC_050144, MT118293
R12	89.5	23	1	65,415	55.5	90	*P. aeruginosa*	[59]	NC_048662, LC472881
Antinowhere	89.0	3	1	65,852	55	92	*P. aeruginosa*	[60]	NC_050150, MT119374
R26	88.3	24	1	65,737	55.5	93	*P. aeruginosa*	[59]	NC_048663, LC472882
F8	87.6	14	1	66,015	55	91	*P. aeruginosa*	[42]	NC_007810, DQ163917
Epa61	87.4	12	1	65,905	55	92	*P. aeruginosa*	---	NC_048744, MK317959
PB1	86.9	21	1	65,764	55.5	93	*P. aeruginosa*	[52]	NC_011810, EU716414
Ab28	86.4	2	1	66,181	55	91	*P. aeruginosa*	[43]	NC_026600, LN610589
DL60	86.1	7	1	66,103	55	89	*P. aeruginosa*	[61]	NC_028745, KR054030
datas	82.5	6	1	60,746	55	89	*P. aeruginosa*	---	NC_050143, MT119378

**Table 3:** Antibiotic susceptibility of *P. aeruginosa* strains susceptible to PaCCP1 or PaCCP2. Colors indicate the interpretation of the zone diameters: resistance (red), intermediate (yellow), or susceptibility (green). Numbers indicate the diameter in mm of the inhibition zone. The strains infected by PaCCP1 (^a^) and PaCCP2 (^b^) are indicated.

Strain	AMK	CIP	MEM	IPM	CZA	CAZ	FEP	TZP
UC522 ^a^	21	25	11	10	25	20	25	23
UC528 ^a^	22	30	12	8	21	12	14	22
UC529 ^b^	20	26	20	12	24	12	6	24
UC532 ^ab^	14	15	6	6	22	16	20	23
UC533 ^a^	22	20	19	20	26	15	22	21
UC535 ^b^	13	19	10	6	21	16	22	23
UC536 ^a^	22	26	29	25	28	26	26	26

Antibiotics: amikacin (AMK), ciprofloxacin (CIP), meropenem (MEM), imipenem (IPM), ceftazidime/avibactam (CZA), ceftazidime (CAZ), cefepime (FEP), and piperacillin/tazobactam (TZP).

**Table 4:** Putative Acr-encoding genes detected in phage PaCCP1 and PaCCP2. The cutoff threshold for PaCRISPR is >0.5, and the cutoff threshold for AcRanker is >−5.0.

CDS	PaCRISP Score	AcRanker Score	Rank by AcRanker	Length (AA)	Sequence
PaCCP1					
41	0.73	−2.57	1	69	MKRNVKVLLAIAAIVAAFGVVGSMDYRDEVREQLSYCENVKNGVWPDFKEWGKTECSPERIAELENILR
33	0.62	−3.01	2	101	MGAYTVTTEFEMNDGRILSCEYGVSFTPGNYSGLPENCYPDESEAGEPTYYIDGEEVDYKDLPKGLDKIADKLYEAGPGEYGYSETEPDYDGPDYEPDDYY
28	0.56	−4.45	3	69	MKRNVKVLLAIAAIVAAFGVVGSMDYRDEVREQLSYCENVKNGVWPDFKEWGKTECSPERIAELENILR
PaCCP2					
39	0.88	−2.66	1	100	MTKQVQIEVTNLDEAFVQHLLTGGHLFDVDDYEVADRILMEVDGEQMVQFELNAELWNEETLGVPMDIDSDEFADELQDWVESKVNFAFEEWLSADEGEE
43	0.52	−3.59	2	64	MNATYQALKTLRDSCEAAKDEKGTINGNKLNALRNKAVKEMEAGGETYSDAIAMAHDLIKKYRK
73	0.59	−4.03	5	123	MAFGVIGTQIVKYRKFEQRVKNDQAQYVSMFEEPFDLAASVQRVRRDQYVQFNLEFQRNYVMIFANFEMVDLDRDVAGDQFLWTGRVFQLESQGSWFYQDGWGVCLAVDIGAAKLTDDGKPTF
35	0.59	−4.39	10	83	MTSSKWTIGRNDTIEVEAVNSREDFRWNGKIRVIHYSAGQIVNIIEFYHHDLDWAIKNFGIKLKAISKGMEILHTCYFGKYVK
36	0.60	−4.51	12	61	MTKAEQLLKARLEDIAAAAVRRERLDWAVSYTFDDGSSIQCRKLSGRSAFARNGWSLGSTD
1	0.79	−4.71	14	68	MSIELVYRTSDGTVFSSVQEAEEYESRLEACELLKEEIEQYGLRKEQAQGLALALTEKFHFTPIPEDF

**Table 5:** Random subsampling of reads used for the assemblies of phages PaCCP1 and PaCCP2.

	Raw Reads		After Random Subsampling (2%)	
	Total Reads (×2)	Total Bases (×2)	Total Reads (×2)	Total Bases (×2)
PaCCP1	1,716,957	249.9 Mbp	34,189	4.9 Mbp
PaCCP2	1,283,165	190 Mbp	25,587	3.7 Mbp

## Data Availability

The sequencing data for bacteriophages PaCCP1 and PaCCP2 are available in GenBank under accession numbers PQ492277 and PQ492278, respectively.
